# Engineering of near infrared fluorescent proteinoid-poly(L-lactic acid) particles for *in vivo* colon cancer detection

**DOI:** 10.1186/s12951-014-0030-z

**Published:** 2014-08-12

**Authors:** Michal Kolitz-Domb, Igor Grinberg, Enav Corem-Salkmon, Shlomo Margel

**Affiliations:** 1 Department of Chemistry, The Institute of Nanotechnology and Advanced Materials, Bar-Ilan University, Ramat-Gan 52900, Israel

**Keywords:** Proteinoid nanoparticles, Fluorescent nanoparticles, NIR fluorescence, Optical imaging, Colon cancer

## Abstract

**Background:**

The use of near-infrared (NIR) fluorescence imaging techniques has gained great interest for early detection of cancer owing to the negligible absorption and autofluorescence of water and other intrinsic biomolecules in this region. The main aim of the present study is to synthesize and characterize novel NIR fluorescent nanoparticles based on proteinoid and PLLA for early detection of colon tumors.

**Methods:**

The present study describes the synthesis of new proteinoid-PLLA copolymer and the preparation of NIR fluorescent nanoparticles for use in diagnostic detection of colon cancer. These fluorescent nanoparticles were prepared by a self-assembly process in the presence of the NIR dye indocyanine green (ICG), a FDA-approved NIR fluorescent dye. Anti-carcinoembryonic antigen antibody (anti-CEA), a specific tumor targeting ligand, was covalently conjugated to the P(EF-PLLA) nanoparticles through the surface carboxylate groups using the carbodiimide activation method.

**Results and discussion:**

The P(EF-PLLA) nanoparticles are stable in different conditions, no leakage of the encapsulated dye into PBS containing 4% HSA was detected. The encapsulation of the NIR fluorescent dye within the P(EF-PLLA) nanoparticles improves significantly the photostability of the dye. The fluorescent nanoparticles are non-toxic, and the biodistribution study in a mouse model showed they evacuate from the body over 24 h. Specific colon tumor detection in a chicken embryo model and a mouse model was demonstrated for anti-CEA-conjugated NIR fluorescent P(EF-PLLA) nanoparticles.

**Conclusions:**

The results of this study suggest a significant advantage of NIR fluorescence imaging using NIR fluorescent P(EF-PLLA) nanoparticles over colonoscopy. In future work we plan to broaden this study by encapsulating cancer drugs such as paclitaxel and/or doxorubicin, within these biodegradable NIR fluorescent P(EF-PLLA) nanoparticles, for both detection and therapy of colon cancer.

## Background

In recent years, several types of nanoparticles have been introduced to the field of cancerous tissue detection. When referring to colon cancer specifically, the early detection of adenomatous colonic polyps is a major concern, as the early detection is the key to survival [1],[2]. Up until now, colorectal cancer screening includes either stool-based tests or endoscopic and radiological examination of the colon [3],[4]. These techniques are considered to be invasive and insensitive, causing poor patient compliance. Thus, colon cancer continues to be a major cause of death. As recently studied and published by several research groups, novel near-infrared (NIR) fluorescent nanoparticles may serve as a valuable tool in the field of colon tumor detection [5],[6]. The nanoparticles introduce the use of fluorescence in the NIR region (700–1000 nm), where autofluoresence, light scattering and absorption of the light by normal tissues is not a concern. This way, the imaging has an improved signal-to-noise ratio, as the background is non-fluorescent in the NIR region and the detection of the fluorophore is optimal [7]–[9]. Nanoparticles containing NIR dyes have already been developed and proved to have significant advantages over free NIR dyes, including biocompatibility, improved fluorescence signal, enhanced photostability and the presence of functional groups on the nanoparticle surface allowing easy conjugation to bioactive molecules [6],[10]–[12]. Among various NIR fluorescent dyes, cyanine dyes are already approved and used in a wide range of biological applications, since they are known as water-soluble, stable, sensitive and have sharp fluorescence bands [13].

Recently, proteinoid particles were studied by several groups as new drug delivery systems [14]–[16]. The thermal condensation of amino acids into proteinoids was first described and characterized by Fox and Harada [17]–[20]. When proteinoids are incubated in an aqueous solution they form hollow particles that range in size according to the environment conditions [21]. Since proteinoid particles are considered biodegradable, non-immunogenic and non-toxic, they can be used as a delivery vehicle in the body [16]. In this work, a new version of proteinoid is synthesized and studied. In order to synthesize suitable particles for this application, proteinoids made of natural amino acids along with low molecular weight poly(L-lactic acid) (PLLA) were synthesized. The natural amino acids L-glutamic acid (E) and L-phenylalanine (F) are thermally polymerized with and without 2000 Da PLLA. All of the monomers used are safe, without exception, as PLLA is widely used in many biomedical applications as medical implants, such as screws, pins, sutures, rods [22],[23], etc.

After preparation, the crude proteinoids can go through a self-assembly process to form micro- and nano-sized particles [24],[25]. If a molecule of a dye or a drug is introduced during the self-assembly, the process may include the encapsulation of the molecule within the proteinoid particle [16]. Here, indocyanine green (ICG), a well-known fluorescent cyanine dye, was encapsulated by the proteinoid-PLLA to form new NIR fluorescent proteinoid-PLLA nanoparticles. Leakage of the entrapped NIR dye into PBS in the absence and the presence of 4% albumin was not detected.

The NIR fluorescent P(EF-PLLA) nanoparticles, containing ICG, were tested for their *in vivo* biodistribution in a mouse model. Additionally, the NIR fluorescent P(EF-PLLA) nanoparticles were conjugated to a bioactive targeting molecule: anti-cacinoembryonic antigen antibodies (anti-CEA) [12]. The bioactive-conjugated NIR fluorescent P(EF-PLLA) nanoparticles were found to specifically detect colon cancer tumors, as demonstrated in tumor implants in a chicken embryo model and in a mouse model.

## Results and discussion

In the first stage of this study P(EF) and P(EF-PLLA) polymers and particles have been prepared and characterized as described in the Materials and methods section. Table 1 compares the physical and chemical properties of the formed polymers and particles.

**Table 1:** Characterization of the P(EF) and P(EF-PLLA) polymers and particles

	**P(EF)**	**P(EF-PLLA)**^**f**^
**Mw (kDa)**^**a**^	165	168
**Mn (kDa)**^**a**^	138	156
**PDI**^**a**^	1.19	1.07
**Optical activity [α]**_**D**_^**25**^**°**^**C**^**(°)**^**b**^	−9.0	−4.5
**Particle diameter (nm)**^**c**^	196 ± 24	103 ± 11
**Particle density (g/mL)**^**d**^	0.001	0.005

^**a**^Molecular masses were measured by GPC, PDI is the polydispersity index, given by Mw/Mn; ^**b**^specific optical rotation (c = 1, in H_2_O, at 25°C); ^**c**^hydrodynamic particle diameter, measured by DLS, as described in the Materials and methods section; ^**d**^particle density was measured as described in the Materials and methods section; ^**f**^P(EF-PLLA), PLLA 10% of the total monomer weight.

The incorporation of low molecular weight PLLA segments (2000 Da) in the copolymer backbone contributes to the overall polymer biodegradability and the smaller nanometric-scale size of the particles made of it. Furthermore, it does not affect significantly the molecular weights, improves slightly the polydispersity and decreases significantly the size and size distribution of the obtained nanoparticles from 196 ± 24 to 103 ± 11 nm. Additionally, the presence of the PLLA segments provides an additional safe way for biodegradation through ester hydrolysis [22]. Hence, P(EF-PLLA) was selected as the better polymer for this specific application.

**Figure 1:**
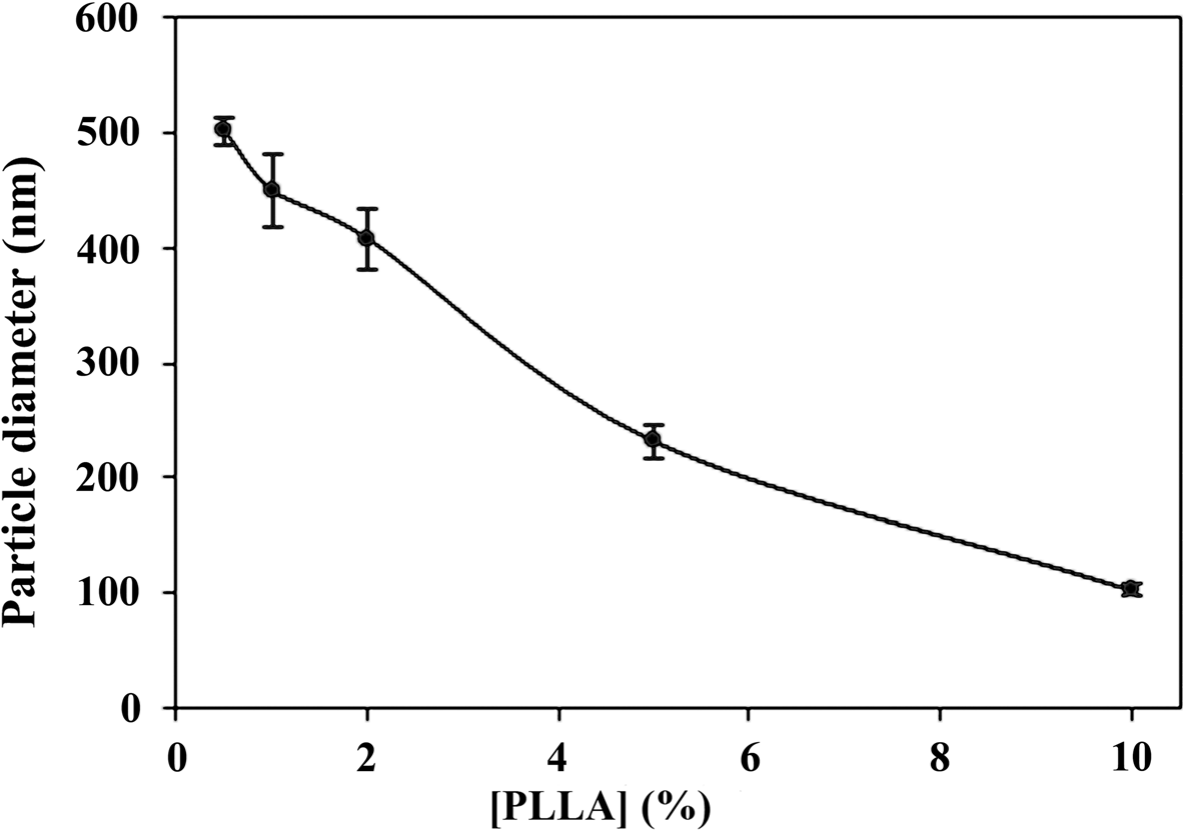
Effect of the PLLA weight% of the total monomer weight in synthesized P(EF-PLLA) on the hydrodynamic particle diameter.

Several P(EF-PLLA) copolymers were synthesized, changing the PLLA percentage in the total monomer weight, as specified in the Materials and methods section. Figure 1 exhibits the effect of changing the PLLA content on the different P(EF-PLLA) particle sizes.

As shown, the optimal particles, judging by their size and size distribution, are the particles made of P(EF-PLLA) where PLLA is 10% of the total monomer. In this case, the particles formed are nanoparticles of the smallest nanometric size with a narrow size distribution, 103 ± 11 nm. When PLLA is of lower percentage in the total monomer weight, the copolymers self-assemble into larger particles in size, 232–502 nm. Overall, as the PLLA fraction in the copolymer rises, the particles formed are smaller in size, indicating the importance of incorporating PLLA into the proteinoid particles, where these hydrophobic moieties are well-packed to form the interior of the nanoparticle. However, P(EF-PLLA) with 20% PLLA does not self-assemble into particles at the specified conditions in the Materials and methods section.

The P(EF-PLLA) nanoparticle density measured as described in the method section was 0.005 g/mL, indicating that the particles formed have a relatively high volume and a very low mass, probably hollow particles, as already known for the proteinoid particles reported in the literature [26]. This is significantly important, since hollow particles may be used for the encapsulation of drugs and dyes, etc.

**Figure 2:**
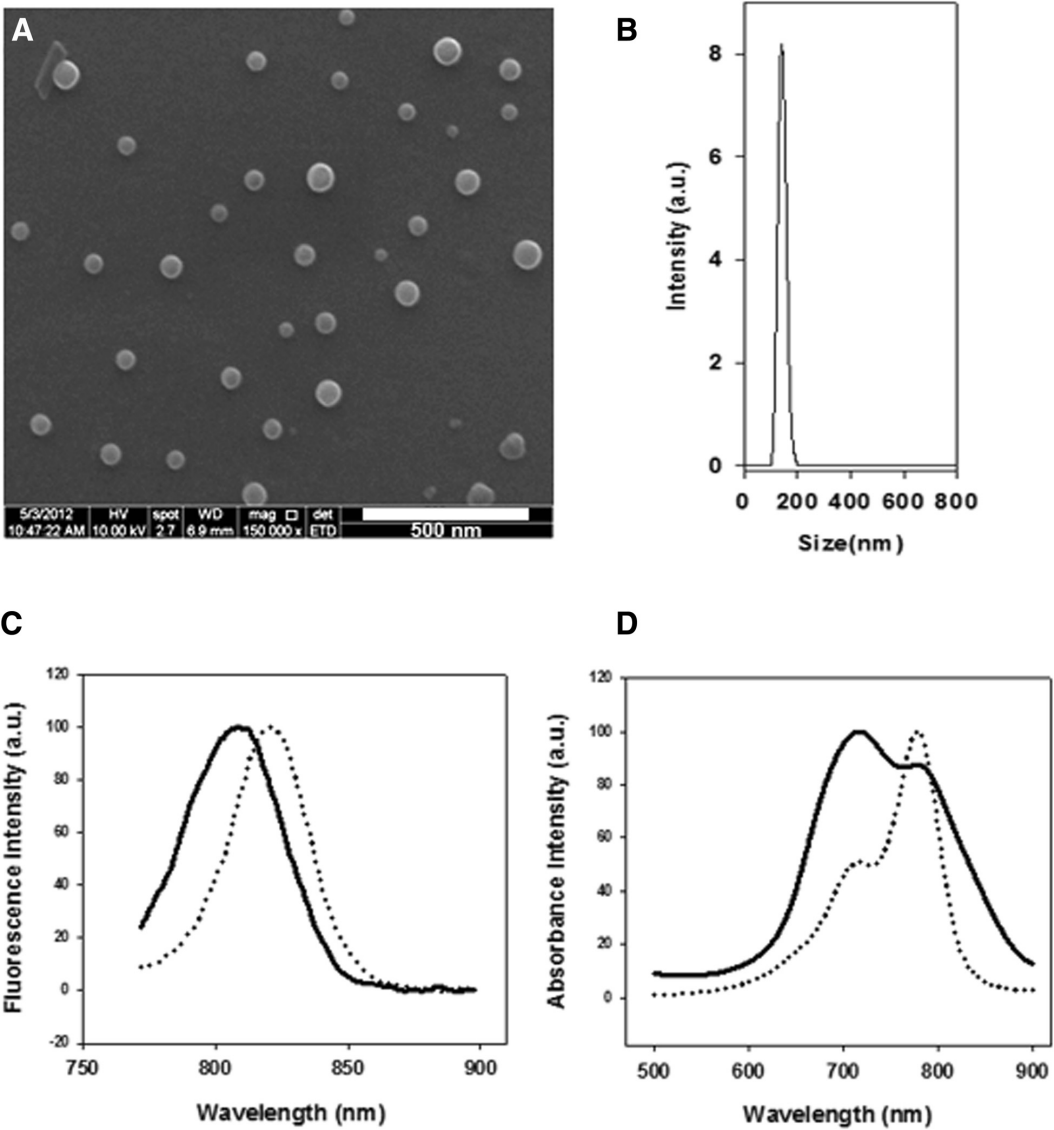
**Characterization of the NIR fluorescent P(EF-PLLA) nanoparticles. (A, B)** SEM image and hydrodynamic size histogram of the NIR fluorescent P(EF-PLLA) nanoparticles; **(C, D)** emission and absorbance spectra of free ICG (dotted lines) and nanoparticles containing ICG (solid lines), respectively.

The optimal P(EF-PLLA) nanoparticles were used to encapsulate ICG. Figures 2A and 2B show that the dry (A) and hydrodynamic (B) diameters of the NIR fluorescent P(EF-PLLA) nanoparticles are 70 ± 15 nm and 145 ± 20 nm, respectively. The difference in diameters is due to the fact that the hydrodynamic diameter takes into account the water molecules around and within the hydrophilic nanoparticles. Figures 2C and 2D exhibit the fluorescence and absorbance spectra of the NIR fluorescent P(EF-PLLA) nanoparticles compared to those of the free dye in solution. The absorbance spectra shows no shift in the absorbance, but a change in the maximal absorbance peak from 779 nm in free ICG to 718 nm in ICG-containing nanoparticles, probably since the ICG molecules get close to each other inside the nanoparticle interior and aggregation may occur and cause this change in absorbance [27],[28]. Moreover, a blue-shift of 12 nm in the emission spectrum of the NIR fluorescent nanoparticles compared to the free ICG in solution was also observed, probably due to the dye molecule aggregation inside the particle.

Leakage of the encapsulated ICG into PBS not-containing and containing 4% albumin at room temperature was not observed, indicating that the dye is strongly associated within the P(EF-PLLA) nanoparticles, probably due to physical interactions between the dye and the polymer.

### Optimization of the ICG concentration encapsulated within the P(EF-PLLA) nanoparticles

In order to optimize the ICG-containing P(EF-PLLA) nanoparticles fluorescence intensity, different concentration (0.5-5% w/w relative to P(EF-PLLA)) of ICG were encapsulated in the P(EF-PLLA) nanoparticles, as described in the Materials and methods section. The concentration of encapsulated ICG that provided the maximum fluorescence intensity of the resultant NIR fluorescent P(EF-PLLA) nanoparticles was 1% w/w relative to P(EF-PLLA). At higher dye concentrations, fluorescence quenching was observed, as the distance between the dye molecules encapsulated within the nanoparticle is shorter, resulting in non-emissive energy transfer between them.

### Photobleaching of the NIR fluorescent P(EF-PLLA) nanoparticles

**Figure 3:**
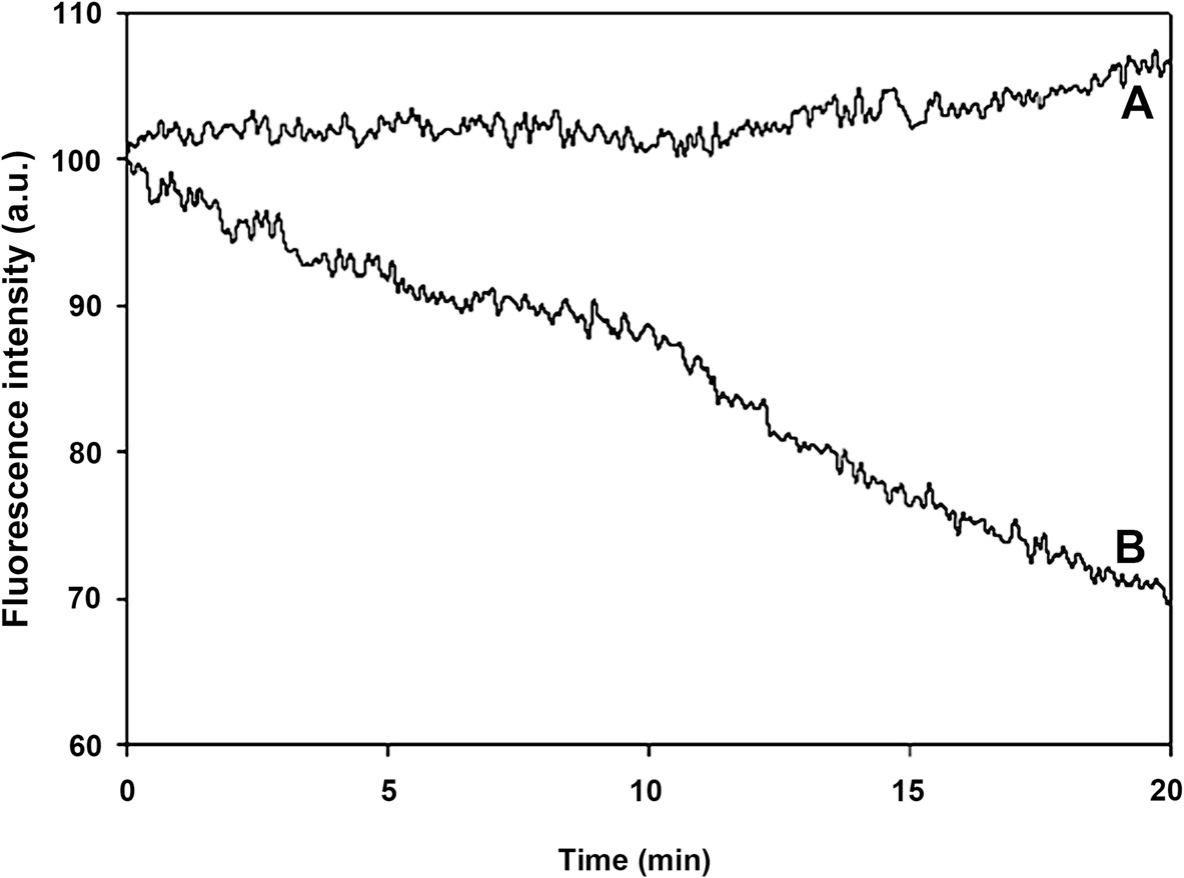
**Photostability of the ICG-containing P(EF-PLLA) particles (A) and free ICG (B) as function of time.** Samples of ICG-containing P(EF-PLLA) particles and free ICG were illuminated with a Xenon flash lamp for 20 min as described in the Materials and methods section.

Photostability experiments of the free and the encapsulated dye within the P(EF-PLLA) nanoparticles were performed. Samples were illuminated at 800 nm and the fluorescence intensities over 20 minutes of illumination were recorded. Figure 3 shows that the fluorescence intensity of free ICG in solution is decreased significantly with time, as opposed to the fluorescence intensity of the encapsulated ICG in the nanparticles, which remains intact. The encapsulation of the dye protects the dye from reactive oxygen species, other oxidizing or reducing agents, temperature, exposure time and illumination levels, which may reduce the fluorescence intensity irreversibly [5],[10].

### Nanoparticle stability

Long-term refrigeration of particle dispersions and freeze-drying of particles are common ways to store nanoparticles for long-time periods. P(EF-PLLA) nanoparticle were kept in these conditions as mentioned in the Materials and methods section, in order to determine their stability. The nanoparticles were kept in refrigeration for 6 months as particle dispersions in PBS, and were found to remain their size and size distribution over this period of time. Additionally, no free ICG or soluble P(EF-PLLA) were detected in the aqueous phase, and the fluorescence intensity of the dispersion remained unaltered. These results indicate that the NIR fluorescent nanoparticles may be kept for long periods of time under refrigeration. The NIR fluorescent nanoparticles were also lyophilized to dryness and then redispersed in an aqueous phase to their original concentration. Yet again, the particles size, size distribution and their fluorescence were not affected. This indicates that the nanoparticles may be stored and handled as a freeze-dried powder and redispersed upon use without the addition of cryoprotectants prior to drying, as common to be done in order to prevent significant agglomeration of nanoparticles [29].

**Figure 4:**
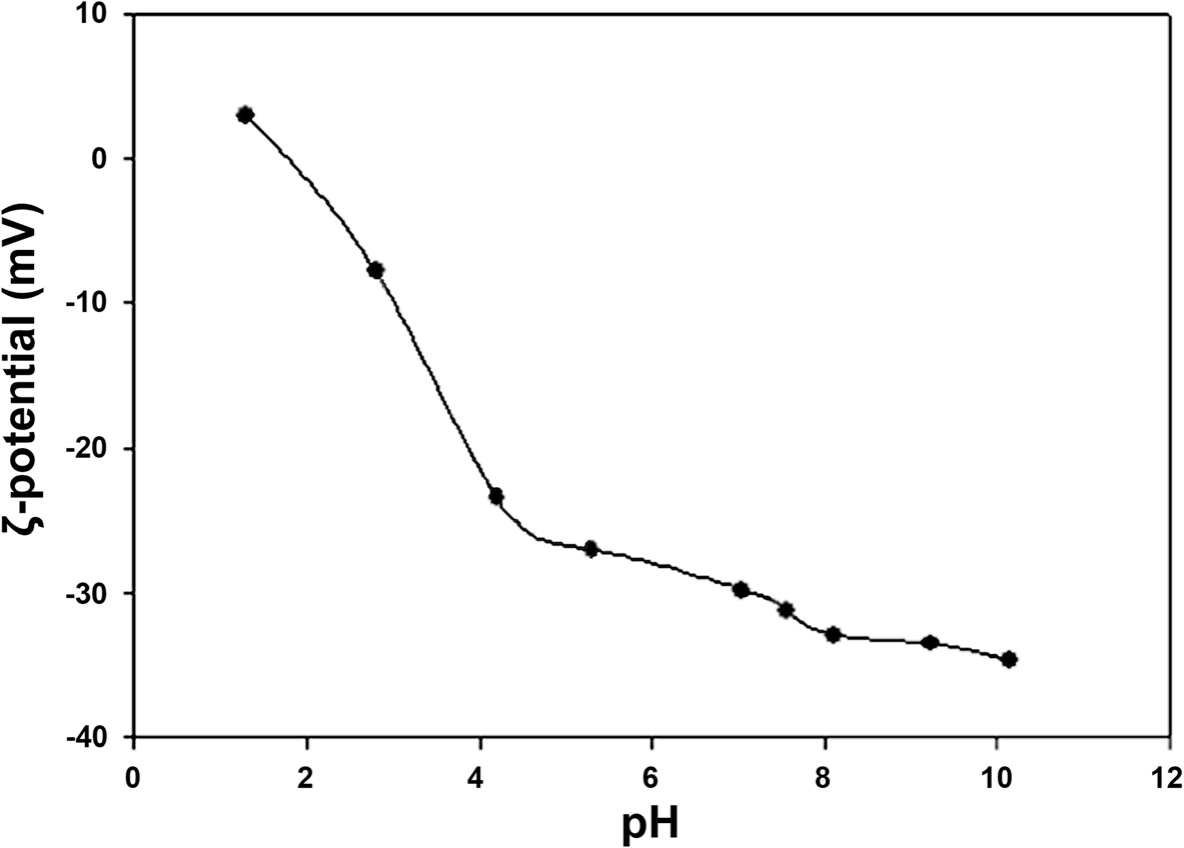
ζ-potential of the NIR fluorescent P(EF-PLLA) nanoparticles as function of pH.

The stability of the fluorescent P(EF-PLLA) nanoparticles as function of pH was tudied by ζ-potential measurements. Figure 4 illustrates the ζ-potential curve of the nanoparticles aqueous dispersion as function of pH. As presented in the figure, there is a decrease in the ζ-potential of the particles as the pH of the particle aqueous dispersions increases: when pH was increased from 1.3 to 10.1, the ζ-potential decreased from 3.0 to −34.7 mV. The isoelectric point of the P(EF-PLLA) nanoparticles is around pH 2, probably due to the large amount of negative charge carboxylate groups that self-assemble on the surface of the nanoparticles.

### *In vitro* cytotoxicity of the P(EF-PLLA) nanoparticles

**Figure 5:**
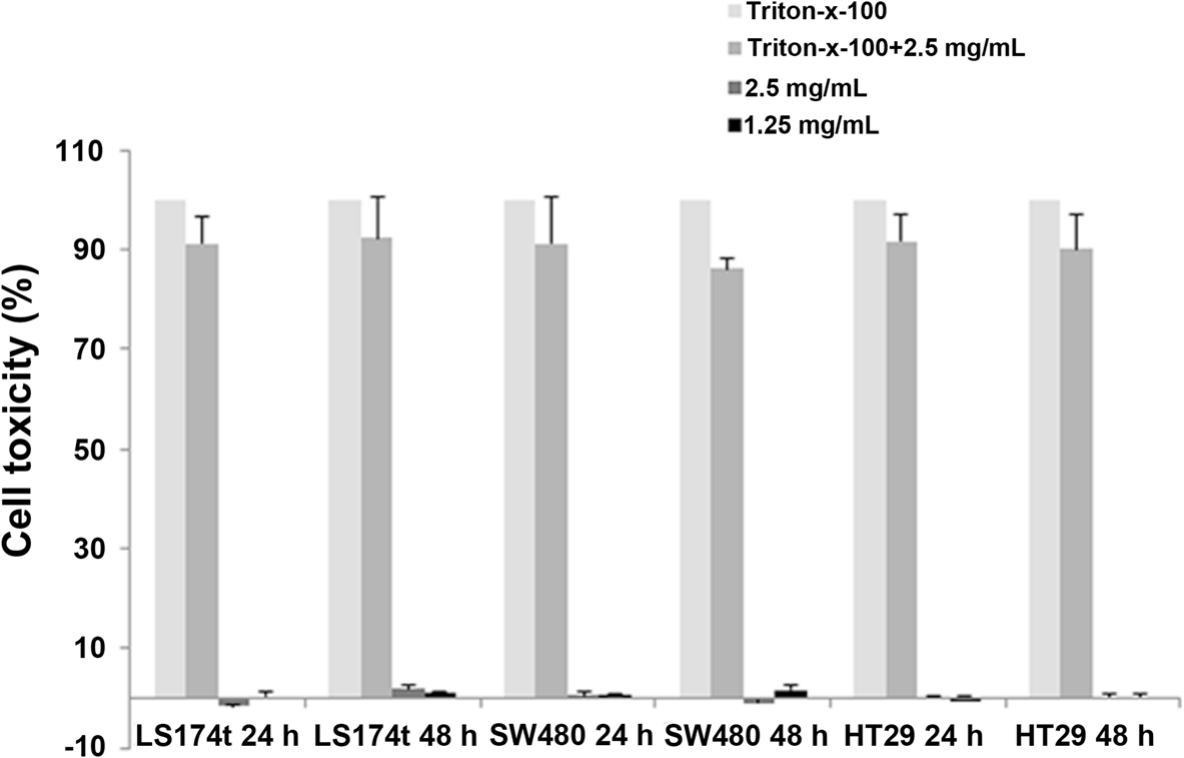
**Cytotoxic effect of the NIR fluorescent P(EF-PLLA) nanoparticles on human colorectal adenocarcinoma LS174t, SW480 and HT29 cell lines measured by the LDH assay.** Cells (3 × 10^5^) were incubated for 24 and 48 h with the P(EF-PLLA) nanoparticles (1.25 and 2.5 mg/mL in PBS). Cells were incubated with 1% Triton-x-100 as positive control (100% toxicity). In addition, cells were incubated with Triton-x-100 1% and the P(EF-PLLA) nanoparticles (2.5 mg/mL) to revoke any interaction of the nanoparticles with the LDH kit components. Untreated cells (negative control) were similarly incubated. Each bar represents mean ± standard deviations of 4 separate samples.

In order to revoke cell toxicity of the NIR P(EF-PLLA) nanoparticles, *in vitro* cytotoxicity of the particles was tested by using human colorectal adenocarcinoma LS174t, SW480 and HT29 cell lines. Cell cytotoxicity was assessed by measuring the release of cytoplasmic lactate dehydrogenase (LDH) into cell culture supernatants. LDH is an intracellular enzyme which catalyzes the reversible oxidation of lactate to pyruvate. Since LDH is predominantly in the cytosol, the enzyme is released into the supernatant only upon cell damage or lysis [30]. Figure 5 exhibits the cytotoxicity levels of the P(EF-PLLA) particles at two different concentrations (1.25 and 2.5 mg/mL). It can be seen that at both concentrations, the P(EF-PLLA) particles have no significant cytotoxic effect on all three cell lines, compared to untreated cells, meaning that the nanoparticles may be used for biomedical applications as suggested, including drug delivery.

### *In vivo* biodistribution in a mouse model

**Figure 6:**
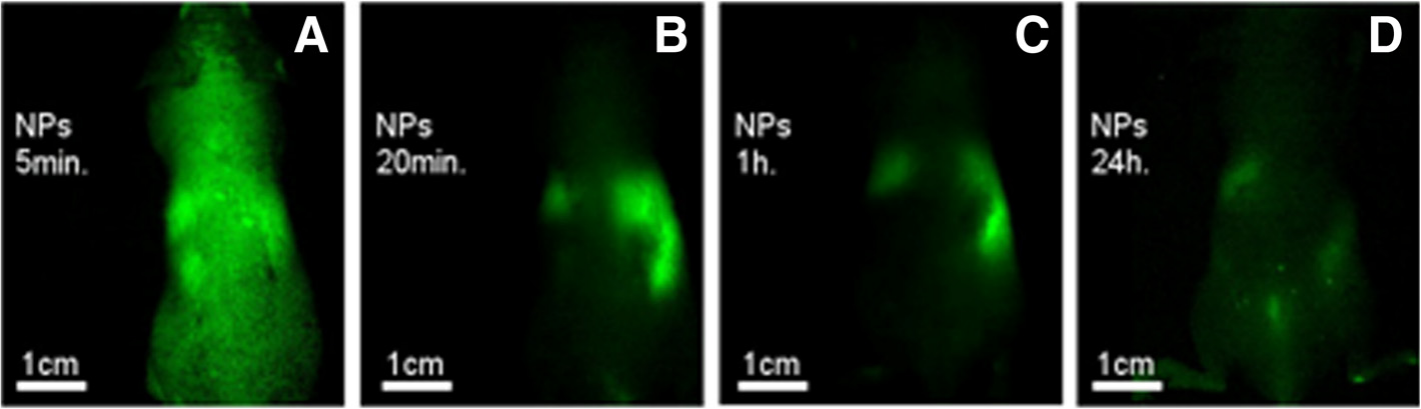
**Typical whole body fluorescence images of the NIR fluorescent P(EF-PLLA) nanoparticles at 5 min (A), 20 min (B), 1 h (C) and 24 h (D) after i.v injection.**12 mice (each experiment group contained 3 mice) were anesthetized and treated with NIR fluorescent P(EF-PLLA) nanparticles (2 mg/mL, 0.01 mg/kg body weight per mouse). Blood was drawn and organs were harvested at each time point. 2 uninjected mice served as negative control. 12 mice were injected correspondingly with free ICG solution, giving similar results (not shown). The experiment was repeated twice with similar results.

NIR fluorescent P(EF-PLLA) nanoparticles (2 mg/mL, 0.01 mg/kg body weight per mouse) were injected i.v. into mice through the tail vein and checked at several time intervals over 24 h. Figure 6 shows whole body images of mice injected with the nanoparticles over time: at 5 min, 20 min, 1 h and 24 h from injection. 5 min post injection, there is an initial burst of fluorescence which subsided quickly, while the majority of the fluorescent nanoparticles concentrated in the liver, at 20 min. 24 h post injection, the fluorescence is almost non-existent, signifying the nanoparticle clearance from the body over 24 h. Biodistribution was tested for free ICG as well, and no significant differences in distribution and kinetics were found between nanoparticles containing ICG and free ICG up to 24 h post injection. These findings were in complete agreement with previous reports of ICG and ICG-containing nanoparticles pharmacokinetics and biodistribution, as the free dye in solution, derivatives of the free dye and ICG-containing nanoparticles are all evacuated from the body after 1 h and completely vanished 24 h after i.v. injection [31],[32].

**Figure 7:**
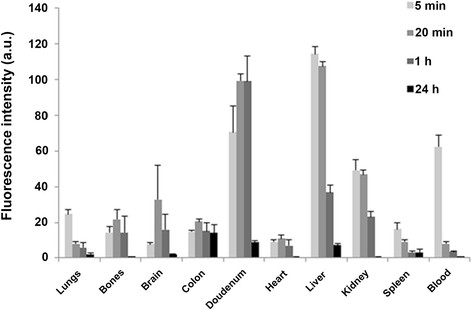
**Fluorescence intensities of different organs taken at 5 min, 20 min, 1 h and 24 h post i.v. injection into mice tail veins.** 12 mice (each experiment group contained 3 mice) were anesthetized and treated with NIR fluorescent P(EF-PLLA) nanoparticles (2 mg/mL, 0.01 mg/kg body weight per mouse). Blood was drawn and organs were harvested at each time point. 2 uninjected mice served as negative control. The experiment was repeated twice with similar results.

*Ex vivo* fluorescence images of specific organs and blood were also obtained. Organs from mice were harvested and blood was drawn 5 min, 20 min, 1 h and 24 h post injection of the nanoparticles into the tail vein. Figure 7 shows the calculated fluorescence intensities of the lungs, bones, brain, colon, duodenum, heart, liver, kidney, spleen and blood screening. Evidently, this analysis shows that the nanoparticles penetrated and were found in all checked organs. It is shown clearly that by 20 min most of the inserted quantity of the fluorescenct nanoparticles is cleared from the blood. The nanoparticles concentrate mostly at the liver and are probably evacuated from the body. Interestingly, it is also apparent that the nanoparticles pass the blood–brain barrier (BBB), since they are found in the brain at 20 min post injection. This may open up a scope of drug targeting to the brain for drug molecules which are usually blocked. Overall, it was demonstrated that following a single i.v. injection of the nanoparticles, fluorescence intensity at all organs decreased over time, and only traces of fluorescence could be seen after 24 h.

### *In vivo* optical detection of human colon tumors on a CAM model

**Figure 8:**
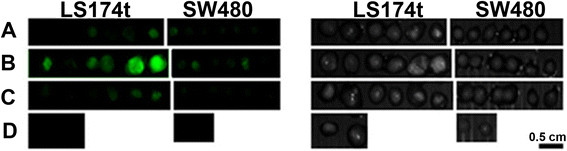
**Fluorescent and grayscale images from a typical experiment of LS174t and SW480 human tumor cell lines implanted on chicken embryo CAM treated with the non-conjugated (A), anti-CEA-conjugated (B) and anti-rabbit IgG-conjugated (C) NIR fluorescent P(EF-PLLA) nanoparticles.** Images of untreated tumors are shown in **(D)**. The experiment was repeated 3 times with similar results.

To demonstrate the feasibility of using the NIR fluorescent P(EF-PLLA) nanoparticles for the detection of colon tumors, monocloneal antibodies against CEA (anti-CEA) and anti-rabbit IgG were conjugated to the nanoparticles. Carcinoembryonic antigen, CEA, is a highly glycosylated glycoprotein expressed in most human carcinomas, and therefore is used as an effective biomarker in several modalities of human carcinoma. As stated in the literature, CEA is upregulated on the mucosal side of the LS174t colorectal cancer cell line, as opposed to SW480 (at least x10^3^ less) [33]. Anti-rabbit IgG was conjugated to nanoparticles as a non-specific binding agent, with the intention of inactivating the conjugated particles in terms of tumor detection. As clearly illustrated in Figure 8, LS174t tumors treated with anti-CEA-conjugated nanoparticles (B) gained greater fluorescence compared to those treated with non-conjugated nanoparticles (A) or anti-rabbit IgG-conjugated nanoparticles (C). This can be explained by the effective ligand-receptor interaction. Furthermore, the SW480 tumors treated with the anti-CEA-conjugated nanoparticles gained less fluorescence (about 3.5 times) compared to the LS174t tumors treated the same way. The fluorescent signal of LS174t tumors labeled by anti-CEA-conjugated nanoparticles was 4 times higher than that of the the tumors labeled by the anti-rabbit IgG-conjugated nanoparticles. Anti-rabbit IgG “blocks” the particle from interacting with the tumor receptors by the conjugation to the surface active moieties, thus serving as a negative control in colon tumor labeling. The results discussed and calculated are an average of 3 different experiments, wherein each experiment contained 6 eggs in a group, altogether 18 eggs in each group.

### *In vivo* optical detection of human colon tumors in a mouse model

**Figure 9:**
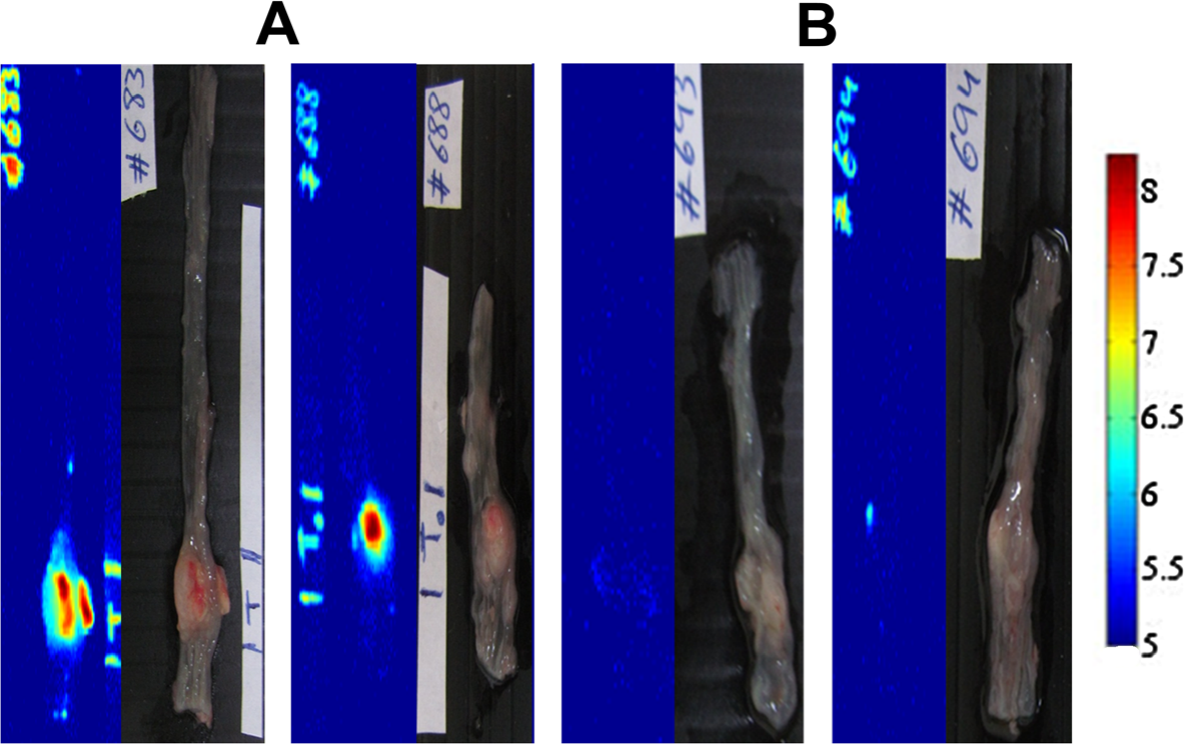
**Fluorescent and grayscale images of typical LS174t colon tumors treated with anti-CEA (A) and anti-rabbit IgG (B) -conjugated NIR fluorescent P(EF-PLLA) nanoparticles.** 20 mice (10 in each experiment group) were anesthetized and treated with 0.1% particle dispersion in PBS, as described in the Materials and methods section. 2 untreated mice served as a control group.

Labeling of human colorectal tumors was performed using orthotopic mouse model (22 mice) with colonic tumors originated from LS174t cells injected to the colon wall 2 weeks before the experiment. Mice were anesthetized and treated with 0.1% bioconjugated NIR fluorescent P(EF-PLLA) nanoparticles dispersion in PBS through the anus. After 20 min, the colons were extensively washed with PBS and were left to recover for 4 h. The colons were then removed and prepared for the imaging as described in the Materials and methods section. Figure 9 shows typical (8 out of 10 mice) fluorescent and grayscale images of the mice colons after treatment with anti- CEA (A) and anti-rabbit IgG (B) conjugated nanoparticles. As illustrated in Figure 9A, the anti-CEA-conjugated nanoparticles detected the tumors specifically and selectively with good signal to background ratio (SBR), the background refers to the surrounding non-pathological tissue. Moreover, as illustrated in Figure 9B, the “inactive” anti-rabbit IgG-conjugated nanoparticles did not produce a significant signal of the tumors.

## Conclusions

Proteinoid and proteinoid-PLLA copolymer were made from (L) glutamic acid, (L) phenylalanine and poly(L-lactic acid) (2 kDa). The optimal copolymer P(EF-PLLA) (10% PLLA), was used to encapsulate ICG to yield NIR fluorescent P(EF-PLLA) nanoparticles. The new NIR fluorescent nanoparticles discussed in the work have the potential to assist in early diagnosis of colonic neoplasms. They are stable, avoiding leakage and photobleaching of the dye over time and non-toxic. The nanoparticles penetrate a variety of organs, including the brain and bones, and are evacuated almost completely over 24 h. The anti-CEA-conjugated NIR fluorescent nanoparticles may be very useful for tumor diagnosis *in vivo*, as they specifically label LS174t colon tumors in the chicken embryo model and in mice.

In future work, we plan to extend the study to include other *in vivo* tumor detection devices, such as whole body imaging or a fluorescent endoscopy camera. Additionally, other tumor-targeting ligands, including antibodies, peptides and proteins (TRAIL and EGF) [34],[35] may be conjugated to the nanoparticles. We also plan to encapsulate anti-cancer drugs such as doxorubicin and/or paclitaxel within the NIR fluorescent P(EF-PLLA) nanoparticles, providing a strategy for both diagnosis and therapy of colon cancer.

## Materials and methods

### Materials

The following analytical-grade chemicals were purchased from commercial sources and were used without further purification: L-glutamic acid (E), L-phenylalanine (F), indiocyaninegreen (ICG), human serum albumin (HSA), bovine plasma fibrinogen, 1-ethyl-3-(3-dimethylaminopropyl) carbodiimide (EDC), Matrigel, Triton-x-100 and monoclonal anti-CEA antibodies (T86-66) from Sigma (Rehovot, Israel); Poly(L-lactic acid) MW 2,000 Da from Polysciences (Warrington, PA, USA); *N*-hydroxysulfosuccinimide (Sulfo-NHS) and 2-morpholino ethanesulfonic acid (MES, pH 6) from Thermo Fisher Scientific (Rockford, IL, USA); Phosphate Buffered Saline (PBS), Minimum Essential Medium (MEM) eagle, McCoy’s 5A medium and Dulbecco’s modification of eagle’s medium (DMEM), Fetal Bovine Serum (FBS), glutamine, penicillin, streptomycin and mycoplasma detection kit from Biological Industries (Bet Haemek, Israel); cell cytotoxicity assay kit (LDH) from Roche (Switzerland); LS174t, SW480 and HT29 cell lines from American Type Culture Collection (ATCC); donkey anti-rabbit IgG from Jackson ImmunoResearch Laboratories (West Grove, PA, USA); water was purified by passing deionized water through an Elgastat Spectrum reverse osmosis system (Elga Ltd., High Wycombe, UK).

### Synthesis and characterization of the P(EF) and P(EF-PLLA) proteinoids

L-glutamic acid (E) was heated at 180°C in an oil bath, under nitrogen atmosphere until melting. The liquefied mass was stirred at 180°C for 30 min. To this, L- phenylalanine (F) and poly(L-lactic acid) (PLLA, Mw = 2000 Da) were added, and kept at 180°C under nitrogen. The total monomer weight was 5 g, PLLA was added at different percentages (1-20% of the total monomer weight) or not added at all. The mixture was mechanically stirred at 150 rpm for 3 h. The product is a highly viscous orange-brown paste, which hardens to give a glassy mass when cooled to room temperature. Then, water (10 mL) was added to the crude product, and the mixture was stirred for 20 min. The solution was then intensively dialyzed through a cellulose membrane (3500 Da MWCO) against distilled water. The content of the dialysis tube was then lyophilized to obtain a yellow-white powder.

The molecular weights and polydispersity indices of the dried P(EF) and P(EF-PLLA) were determined using Gel Permeation Chromatography (GPC) consisting of a Waters Spectra Series P100 isocratic HPLC pump with an ERMA ERC-7510 refractive index detector and a Rheodyne (Coatati, CA) injection valve with a 20 μL loop (Waters, MA). The samples were eluted with super-pure HPLC water through a linear BioSep SEC-s3000 column (Phenomenex) at a flow rate of 1 mL/min. The molecular weight was determined relative to poly(ethylene glycol) standards (Polymer Standards Service-USA, Silver Spring, MD) with a molecular weight range of 100–450000 Da, Human Serum Albumin (HSA, 67 kDA) and bovine plasma fibrinogen (340 kDa), using Clarity chromatography software. The optical activities of the P(EF-PLLA) and P(EF) were determined using a PE 343 polarimeter (PerkinElmer). The measurements were done in water, at 589 nm at 25°C.

### Synthesis of the non-fluorescent and NIR fluorescent P(EF) and P(EF-PLLA) nanoparticles

The P(EF) and P(EF-PLLA) nanoparticles were prepared by a self-assembly process [21]. Briefly, 100 mg of the dried fabricated P(EF) or P(EF-PLLA) were resuspended in 10 mL of 10^−5^ N NaCl solution. The mixture was then heated to 80°C while stirring for 15 min. The mixture was removed from the hot plate and was allowed to return to room temperature. During the cooling process, particles formed and precipitated from the aqueous solution. The formed nanoparticles were then dialyzed versus 4 L of 10^−5^ NaCl solution overnight at room temperature.

NIR fluorescent particles P(EF-PLLA) nanoparticles were prepared by the same procedure, with the addition of ICG (1 mg, 1% of the copolymer) to the hot solution, prior to particle formation.

### Characterization of the non-fluorescent and NIR fluorescent P(EF) and P(EF-PLLA) nanoparticles

Particle size and size distribution were determined using DLS with photon cross-correlation spectroscopy (Nanophox particle analyzer, Sympatec GmbH, Germany) and by Scanning Electron Microscopy (SEM, JOEL, JSM840 Model, Japan). In SEM samples, the diameters of more than 200 particles were measured with AnalySIS Auto image analysis software (Soft Imaging System GmbH, Germany). The density of the particles was determined by pycnometry. Briefly, dry pre-weighed particles were put in a calibrated pycnometer, which was then filled with water. The density of the particles was calculated from the known density of the water, the weight of the pycnometer filled only with water, the weight of the pycnometer containing both the sample and water, and the weight of the sample, as described in the literature [36]. Absorbance spectra were obtained using a Cary 100 UV-Visible spectrophotometer (Agilent Technologies Inc.). Excitation and emission spectra were recorded using a Cary Eclipse spectrofluorometer (Agilent Technologies Inc.). ζ-potential measurements were performed by gradual titration of the nanoparticles’ aqueous dispersion at pH range from 11 to 2.5 using 1 M HCl (Zetasizer zeta potential analyzer 3000 Has Model, Malvern Instruments, England).

### Determination and optimization of the encapsulated ICG concentration in the NIR fluorescent P(EF-PLLA) nanoparticles

The encapsulated ICG concentration was determined for 1 mg/mL nanoparticles, using a calibration curve of the integrals of absorbance peaks of standard free ICG solutions in PBS at 630–900 nm.

Different ICG quantities were encapsulated within the P(EF-PLLA) nanoparticles at weight% ratios of 0.5, 1, 2, and 5% relative to the P(EF-PLLA). The nanoparticle dispersions were diluted to 1 mg/mL in PBS and their fluorescence intensities at 809 nm were measured.

### Leakage extent of the encapsulated ICG from the nanoparticles dispersed in PBS containing 4% HSA

NIR fluorescent P(EF-PLLA) nanoparticles dispersions (1 mg/mL in PBS containing 4% HSA) was shaken at 37°C for 24 h and then filtered via a 300-kDa filtration tube (VS0241 VIVA SPIN) at 4000 rpm (Centrifuge CN-2200 MRC). The fluorescence intensity of the supernatant was then measured at 809 nm.

### Photostability of the NIR fluorescent P(EF-PLLA) nanoparticles

The photostability of the NIR fluorescenet P(EF-PLLA) nanoparticles was examined by recording the fluorescence intensity over a period of 20 min of nanoparticle dispersion in PBS and a PBS solution of free ICG (0.05 M). The nanoparticle dispersion was diluted to give comparable fluorescence intensity to the ICG solution, with λex set at 780 nm and λem set at 800 nm. The excitation and emission slits were opened to 20 nm and 5 nm, respectively. Each of the samples was illuminated continuously with a xenon lamp, and the fluorescence intensity was recorded using a Cary Eclipse fluorescence spectrophotometer (Agilent Technologies Inc.). Intensity values were normalized for comparison.

### Particle stability in storage conditions

NIR fluorescent P(EF-PLLA) nanoparticles aqueous dispersions (1 mg/mL) were put in a refrigerator at 4°C for 6 months. Samples were taken at different time periods, filtered through a centrifugation tube (Vivaspin 3000 Da MWCO) and the filtrate was checked by UV at 200–210 nm, to find soluble P(EF-PLLA) and at 630–900 nm to find free ICG. The nanoparticle size, size distribution and fluorescence were checked as well by the same procedures mentioned above.

Additionally, the nanoparticles were freeze-dried in order to check their stability after drying. Following lyophilization, the nanoparticles were resuspended in PBS to their original concentration and the dispersions were retested for particle size, size distribution and fluorescence intensity.

### *In vitro* cytotoxicity of the NIR fluorescent P(EF-PLLA) nanoparticles

In vitro cytotoxicity of the P(EF-PLLA) nanoparticles was tested by using human colorectal adenocarcinoma LS174t, SW480 and HT29 cell lines. The cell lines are adherent to the used culture dishes. LS174t cells were grown in Minimum Essential Medium (MEM) eagle supplemented with heat-inactivated fetal bovine serum (FBS, 10%), penicillin (100 IU/mL), streptomycin (100 μg/mL) and L-glutamine (2 mM). SW480 cells were maintained in Dulbecco’s MEM supplemented with heat-inactivated fetal bovine serum (FBS, 10%), penicillin (100 IU/mL), streptomycin (100 μg/mL) and L-glutamine (2 mM). HT29 cells were maintained in McCoy’s 5A medium supplemented with FBS (10%), penicillin (100 IU/mL), streptomycin (100 μg/mL) and L-glutamine (2 mM). Cells were screened to ensure they remained mycoplasma-free using Mycoplasma Detection Kit [37].

Cell cytotoxicity was assessed by measuring the release of cytoplasmic lactate dehydrogenase (LDH) into cell culture supernatants. LDH activity was assayed using the Cytotoxicity Detection Kit according to the manufacturer’s instructions [30]. Cells (3 × 10^5^ cells per well) were seeded and grown to 90–95% confluency in 24 well plates before treatment with the P(EF-PLLA) nanoparticles. Cell cultures that were not exposed to the nanoparticles were included in all assays as negative controls. Cell cultures that were treated with 1% Triton-x-100 were used as positive controls. To test if the nanoparticles can interact with LDH kit compounds, cell cultures were exposed to a mixture containing maximal nanoparticles concentration (2.5 mg/mL) dispersed in PBS and 1% Triton-x-100.

The P(EF-PLLA) nanoparticles were freshly dispersed in PBS (1.25 and 2.5 mg/mL) and then added to the 95% confluent cell culture in culture medium. The cell cultures were further incubated at 37°C in a humidified 5% CO_2_ incubator and then checked for cellular cytotoxicity at intervals of 24 h. The percentage of cell cytotoxicity was calculated using the formula shown in the manufacturer’s protocol [30]. All samples were tested in tetraplicates.

### Biodistribution in a mouse model

Male BALB/C mice (Harlan Laboratories, Israel) were utilized in this study under a protocol approved by the Institutional Animal Care and Use Committee at Bar-Ilan University. The biodistribution of the NIR fluorescent P(EF-PLLA) nanoparticles was studied in normal 8-weeks-old mice, weighing 20–25 g at the time of experiment. Prior to the experiment, mice were anesthetized by intraperitoneal injection of Ketamine (40–80 mg/kg body weight) and Xylazine (5–10 mg/kg body weight), and the mice’s skin was shaved with an electric animal clipper.

100 μL of either nanoparticle dispersion or free ICG solution (0.01 mg/kg body weight, dissolved in PBS) were administered to the mice through tail vein injection at a concentration of 2 mg/mL. During image acquisition, mice remained anesthetized by the intraperitoneal injection of Ketamine/Xylazine. Image cubes were obtained from the mice at several time points up to 24 h after injection. Each treatment group includes 3 mice for each time point (5 min, 20 min, 1 h and 24 h); 2 uninjected mice served as negative control. The experiment was repeated twice, testing a total of 52 mice. At the end of the experiment, the mice were euthanized by cervical dislocation, and organs were taken for imaging (liver, spleen, kidney, duodenum, colon, brain, heart, tibia bone and blood).

Whole body fluorescence images were acquired using a Maestro II *in vivo* fluorescence imaging system (Cambridge Research &Instrumentation, Inc., Woburn, MA). The system is equipped with a fiber-delivered 300 W xenon excitation lamp, and images can be acquired from λ = 500-950 nm by a 1.3 megapixel CCD camera (Sony ICX285 CCD chip). Each pixel within the image cube therefore has an associated fluorescence spectrum. The software for the Maestro system (Maestro 2.10.0) contains several algorithms to process the spectral data cubes to remove undesired auto-fluorescence signal and generate overlaid images for multiple fluorophores. A deep red excitation/emission filter set was used for our experiments (λex: 700–770 nm, λem > 780 nm). The liquid crystal tunable filter (LCTF) was programmed to acquire image cubes from λ = 780 nm-860 nm with an increment of 10 nm per image. The camera was set to 150 ms (whole body image), 15 ms (liver), 500 ms (spleen), 7000 ms (kidney), 10 ms (duodenum), 500 ms (colon), 1000 ms (brain), 1000 ms (tibia bones), 200 ms (heart) and 1000 ms (blood) exposure times. Fluorescence intensity measurements were performed using ImageJ NIH (National Institutes of Health) software.

### Conjugation of tumor-targeting ligands to the NIR fluorescent P(EF-PLLA) nanoparticles

Anti-CEA was covalently conjugated to the nanoparticles through carbodiimide activation of the carboxylate groups on the particle surface [6]. Briefly, EDC (1 mg) and Sulfo-NHS (1 mg) were dissolves separately in 1 mL 0.1 M MES containing 0.5 M NaCl. The EDC solution (10 μL, 1 mg/mL) was then added to an aqueous anti-CEA solution (62.5 μL, 0.25 mg) followed by the addition of the Sulfo-NHS solution (25 μL, 1 mg/mL). The mixture was shaken at room temperature for 15 min, and then the NIR fluorescent nanoparticle dispersion was added (2.5 mg in 1 mL PBS). The mixture was shaken for an additional 90 min. The obtained anti-CEA-conjugated fluorescent nanoparticles were then washed from excess reagents by dilution and filtration through a 30-kDa filtration tube (VS2021 VIVA SPIN) at 1000 rpm (Centrifuge CN-2200 MRC) for 2 min, repeated three times. Anti-rabbit IgG was conjugated to the NIR fluorescent nanoparticles through a similar procedure. The concentration of bound anti-CEA and anti-rabbit IgG (1.9 ± 0.2 μg/mg nanoparticles) was determined using a mouse IgG ELISA kit (Biotest, Israel).

### Optical detection of human colon tumor with the non-conjugated and bio-conjugated NIR fluorescent P(EF-PLLA) in a chicken embryo chorioallantoic membrane (CAM)

Human colorectal adenocarcinoma LS174t and SW480 cell lines were used for each of the experiments and maintained as mentioned above. Tumor cells were grafted on CAM according to the literature [38]. Briefly, fertile chicken eggs obtained from a commercial supplier were incubated at 37°C at 60–70% humidity in a forced-draft incubator. On day 3 of incubation, an artificial air sac was formed, allowing the CAM to drop. After 8 days of incubation, a window was opened in the shell and the CAM was exposed. Tumor cells were collected by trypsinization, washed with culture medium and pelleted by gentle centrifugation. Following removal of the medium, 5×10^6^ cells were resuspended in 30 μL ice-cold Matrigel and inoculated on the CAM at the site of the blood vessels. Eggs were then sealed and returned to incubation. On day 6 post-grafting, day 14 of incubation, the tumor diameter ranged from 3 to 5 mm with visible neoangiogenesis.

Chicken embryos with 6-days-old human adenocarcinoma tumors (LS174t and SW480 cancer cell lines) implanted on the CAM were treated with the non-conjugated, anti-CEA-conjugated and anti-rabbit IgG-conjugated NIR fluorescent P(EF-PLLA) nanoparticles (40 μL, 2 mg/mL). Additionally, non-pathological CAM treated with nanoparticles and untreated tumors served as control groups. After 40 minutes, the nanoparticle dispersions were removed and the tumors were washed with PBS. Then, the tumors and the non-pathological CAM were removed from the eggs, washed again with PBS and spread on a mat black background for observation using a Maestro II *in vivo* imaging system (Cambridge Research & Instrumentation, Inc., Woburn, MA). A NIR excitation/emission filter set was used for the experiments (λex: 710–760 nm, λem > 750 nm). The Liquid Crystal Tunable Filter (LCTF) was programmed to acquire image cubes from λ = 790 nm to 860 nm with an increment of 10 nm per image. Fluorescence intensity measurements were calculated as average intensity over the tumor surface area, using ImageJ software.

### Optical detection of human colon tumor with the non-conjugated and bio-conjugated NIR fluorescent P(EF-PLLA) in a mouse model

Experiments were performed according to the protocols of the Israeli National Council for Animal Experiments by Harlan Biotech, Israel. Cancerous cells (30 μL containing 2 × 10^6^ LS174t cells) were injected into the mouse intestinal wall. 2 weeks later the nude mice were anaesthetized and treated with the bio-conjugated NIR fluorescent P(EF-PLLA) nanoparticles (0.1%, 200 μL), through the anus, using the guidance of a mini-colonoscope. 20 min later each colon was washed with PBS (5 × 1 mL) and mice were allowed to recover for 4 h. The mice were sacrificed and the colons were removed. Each colon was spread on a solid surface and imaging was performed using the Odysey Infrared Imaging System (Li-Cor Biosciences, Lincoln, NE, USA) with excitation wavelength of 780 nm and emission wavelength of 800 nm.

## Competing interests

The authors declare that they have no competing interests.

## Authors’ contributions

MKD carried out the synthesis and characterization of the nanoparticles. IG and ECS carried out the biological studies, including toxicity assays and the *in vivo* assays. SM supervised the study, and participated in it’s the design and coordination. All authors read and approved the final manuscript.
